# The Avatar’s Gist: How to Transfer Affective Components From Dynamic Walking to Static Body Postures

**DOI:** 10.3389/fnins.2022.842433

**Published:** 2022-06-15

**Authors:** Paolo Presti, Davide Ruzzon, Gaia Maria Galasso, Pietro Avanzini, Fausto Caruana, Giovanni Vecchiato

**Affiliations:** ^1^Institute of Neuroscience, National Research Council of Italy, Parma, Italy; ^2^Department of Medicine and Surgery, University of Parma Parma, Italy; ^3^TUNED, Lombardini22, Milan, Italy; ^4^Dipartimento Culture del Progetto, University IUAV, Venice, Italy

**Keywords:** virtual reality, valence, arousal, dynamic walking, body posture

## Abstract

Dynamic virtual representations of the human being can communicate a broad range of affective states through body movements, thus effectively studying emotion perception. However, the possibility of modeling static body postures preserving affective information is still fundamental in a broad spectrum of experimental settings exploring time-locked cognitive processes. We propose a novel automatic method for creating virtual affective body postures starting from kinematics data. Exploiting body features related to postural cues and movement velocity, we transferred the affective components from dynamic walking to static body postures of male and female virtual avatars. Results of two online experiments showed that participants coherently judged different valence and arousal levels in the avatar’s body posture, highlighting the reliability of the proposed methodology. In addition, esthetic and postural cues made women more emotionally expressive than men. Overall, we provided a valid methodology to create affective body postures of virtual avatars, which can be used within different virtual scenarios to understand better the way we perceive the affective state of others.

## Introduction

The rapid development of virtual technologies makes it possible to investigate human behavior in fictive environmental and social scenarios, which are otherwise difficult to reproduce and study within standard laboratory settings (Sanchez-Vives and Slater, 2005; Slater and Sanchez-Vives, 2016; Presti et al., 2021). In this context, the dynamic information of body kinematics allows virtual representations of human behavior with specific emotional contents. Researchers showed body kinematics provide significant recognition accuracy of emotions (Atkinson et al., 2004), and postural information allows the discrimination of emotion intensity (Aviezer et al., 2012), demonstrating that body cues are fundamental for a comprehensive understanding of the other’s emotion (de Gelder, 2006, 2009; de Gelder et al., 2010). The possibility to model virtual static bodily configurations preserving a sense of dynamicity and affective information is still of particular interest, given the predominant use of static bodily expressions in a broad spectrum of experimental settings that explore time-locked cognitive processes such as event-related potential with electroencephalography and evoked potential with transcranial magnetic stimulation and reaction times in behavioral studies. Moreover, virtual environments without a realistic depiction of human behavior can be uninteresting, resulting in a lack of attention, often required to study cognitive processes in social scenarios. Creating an expressive virtual character is difficult because of the complex nature of human non-verbal behavior, such as body posture, and there is surprisingly little research on models that generate affective behavior (Coulson, 2004; de Gelder, 2006; Vinayagamoorthy et al., 2008). Therefore, the issue to solve is identifying bodily variables that can be used to build a model of affective behavioral cues.

A strategy to model the posture of a virtual avatar with emotional content involves using motion capture technologies to record an actor’s movement and then select the most expressive posture based on subjective judgment (De Silva and Bianchi-Berthouze, 2004; Kleinsmith et al., 2006). Another way is to exploit 3D modeling software to artificially shape the avatar posture by changing the angles between contiguous body parts (Clavel et al., 2009; Buisine et al., 2014). Other systems characterizing emotional expressions depend on the degree of subjective inference and granularity of the measure, whose combination deeply impacts the reliability and efficiency of the categorization in terms of time coding. For instance, the Body Action and Posture Coding System consists of 141 behavioral variables, whose combination describes anatomical articulation, form, and functional level of the movement. A reliability study reports that it took 2,280 min to encode the data set of 6.28 min, thus yielding a coding ratio of 1:363 (Dael et al., 2012). The Facial Action Coding System consists of 44 Action Units that can be coded at different levels of intensity with a coding ratio of 1:100 (Cohn et al., 2007). The Laban Movement Analysis describes how the observed motor action uses components of movement and how each component of movement is related to one another with a coding ratio of 1:30 (Bernardet et al., 2019). As these procedures are highly subjective and time-consuming, there is the need to identify an automatic procedure to transfer the affective information from a body kinematic to a static emotional posture. Hence, this study aims to overcome reliability and time coding issues by automatically extracting body features from kinematic data of walking to transfer the corresponding dynamic affective components to static body postures.

Walking is a natural day-to-day motion that can convey different affective states by combining upper- and lower-limb movements (Montepare et al., 1987; Roether et al., 2009; Karg et al., 2010; Zhao et al., 2019). Previous works have shown that virtual representations of human beings can communicate different affective states through emotional walking (McHugh et al., 2010; Hicheur et al., 2013; Randhavane et al., 2019a,^2021^). A recent study of Bhattacharya et al. (2020) found that participants correctly recognized the emotion expressed by virtual avatars according to different gait patterns. Similar results were found in a study where emotional walking was used to animate virtual avatars in different virtual scenarios (e.g., a park, street, and garden) (Randhavane et al., 2019b). Previous studies in kinematic-based movement analysis and affective computing returned that both postural and kinematic features are essential for an accurate description of the individual’s affective states (Kleinsmith and Bianchi-Berthouze, 2013; McColl and Nejat, 2014; Stephens-Fripp et al., 2017). In this regard, valence and arousal are typically associated with different body features and are considered crucial characteristics to describe the human affective experience on continuous and dimensional scales (Lindquist et al., 2012; Kuppens et al., 2013; Kragel and LaBar, 2016). Valence dimension is described by postural cues defining the body’s shape during the movement. Hence, joint angles between contiguous body segments and the position assumed by specific body parts are crucial cues for identifying the valence level conveyed by the movement. Head and trunk orientation discriminate between positive and negative valence levels. Walking with a downward leaning of the head/trunk highlights unpleasant affective states as such posture is associated with sadness and anger. On the other hand, an upward orientation identifies joyful walking (Karg et al., 2010; Hicheur et al., 2013; Venture et al., 2014; Crenn et al., 2016; Randhavane et al., 2019b). The volume calculated from the expansion of the body in the three-dimensional space is another postural feature widely exploited for the affective characterization of walking. Instead, a compact posture is associated with sad walking, while an expanded one stands for positive expressions (Crenn et al., 2016; Randhavane et al., 2019b). The dimension of arousal is well-described by kinematic cues considering the quantity of motion of the gesture, which is highly correlated with velocity, acceleration, and jerk of the movement (Karg et al., 2010; Sanghvi et al., 2011; Randhavane et al., 2019b) (Nakagawa et al., 2009). Previous studies reported that walking speed is correlated with the arousal level (Roether et al., 2009; Halovic and Kroos, 2018a; Deligianni et al., 2019). Sad walking was characterized by slow movements, while joy and anger walking, typically considered high arousal emotions, were characterized by fast and rapid movements (Bernhardt and Robinson, 2007; Gross et al., 2012; Barliya et al., 2013; Randhavane et al., 2019b).

This study provides a procedure enabling the automatic creation of emotional body postures by identifying the corresponding most salient time frame from a whole kinematic. For this purpose, emotional walking kinematics were described frame per frame by two distinct body features, namely, body pleasantness (BP) and body dynamicity (BD), which were based on the combination of postural cues and movement velocity of male and female actors.

We hypothesize that the time frames automatically selected with different levels of BP and BD should correspond to coherent valence and arousal levels, separately, reflecting those perceived in the corresponding walking actions.

Results of a first experiment showed that participants coherently assigned valence and arousal scores to avatars’ body postures with different levels of BP and BD, and that such scores were strongly correlated with those provided on the correspondent walking actions. Findings also showed that the female avatar was judged as more emotionally expressive than the male one. With the hypothesis that the avatar’s physical appearance could contribute to the judgment of valence and arousal, we performed a second online experiment by disrupting the coherence between the gender of the actor and that of the avatar. Hence, the male avatar assumed those body postures derived from female actresses and *vice versa*. This experiment revealed that the combination of both esthetic and postural characteristics makes women appear more emotionally expressive than men.

Overall, we demonstrated that the proposed methodology successfully transferred affective components from emotional walking to static body postures. The use of BP and BD allowed to select representative emotional frames in a reliable and time-efficient way, leading to the automatic creation of affective body postures. The proposed procedure could be exploited to design stimuli for a broad range of experimental studies investigating time-locked cognitive processes related to the perception of emotional body postures.

## Materials and Methods

### Emotional Kinematics

We considered the EMILYA database to select emotional kinematics for this study (Fourati and Pelachaud, 2014, 2018). Such a database includes daily actions performed by actors and recorded through inertial motion capture technology. We considered the 912 simple walking actions comprising the whole body’s movement of five female and five male actors. A.mat file (MATLAB, The MathWorks, Inc., Natick, MA, United States) contains the time-varying root-related positions of 28 body joints for each action. They refer to a 3D space where the xy, yz, and xz planes describe the coronal, sagittal, and transverse planes, respectively, and the origin of the axes corresponds to the middle point between the right and left hip.

### Body Pleasantness

The valence dimension is generally linked to postural features more than kinematic ones (Nakagawa et al., 2009). Previous findings show that bowing and expansiveness of the body discriminate the pleasantness of the performed action (Karg et al., 2010; Fourati and Pelachaud, 2015, 2018). Here, we defined BP as the feature adopted to transfer the valence information of the walking to a static body posture. Such score was computed as a combination of three distinct body features, namely, the leaning of the head (LH), the position of the head (PH), and the openness of the body (OB), according to the following procedure. For each kinematic, the LH was computed as the time-average distance between the body joints representing the head and the neck in the sagittal direction:


(1) $$\bar{L\,H}=\frac{\sum_{t=1}^{F}\left(p_{t,z\,h\,e\,a\,d}-p_{t,z\,n\,e\,c\,k}\right)}{F}$$

where *p*_*t,  z  head *_ indicates the z coordinate at time t of the body joint related to the head, and *p*_*t,  z  neck *_ indicates the z coordinate at time t of the body joint related to the neck, and F stands for the total number of recorded frames for that kinematic.

The PH was computed as the time-average distance of the body joint representing the head and the origin on the z-axis:


(2) $$\bar{P\,H}=\frac{\sum_{t=1}^{F}\left(p_{t,z\,h\,e\,a\,d}\right)}{F}$$

Finally, the openness of the body was defined as the time-average body spatial extension in the transverse, sagittal, and coronal plane and computed as follows:


(3) $$\bar{O\,B}=\frac{\sum_{t=1}^{F}\left[\left(\max p_{t,x}-\min p_{t,x}\right)*\left(\max p_{t,y}-\min p_{t,y}\right)*\left(\max p_{t,z}-\min p_{t,z}\right)\right]}{F}$$

where max *p*_*t, x*_−min *p*_*t, x*_ stands for the maximum extension of the body in the lateral direction, max *p*_*t, y*_−min *p*_*t, y*_ for the maximum extension in the vertical direction, and max *p*_*t, z*_−min *p*_*t, z*_ stands for the maximum extension of the body in the sagittal direction, each one considered at time *t*.

To describe the valence dimension of the kinematic with a unique value combining the information conveyed by the abovementioned body features, we performed a principal component analysis (PCA) (Abdi and Williams, 2010) using the LH, PH, and OB variables as inputs. We extracted the first principal component explaining the 83% of data variance and defined the BP as the weighted sum of the three input variables. Hence, for each kinematic, we were able to compute the BP frame by frame according to the following formula:


(4) $$B\,P_{t}=0,77*L\,H_{t}+0,63*P\,H_{t}+0.10*O\,B_{t}$$

and then select the frame which BP score was the closest to the mean BP of the whole kinematic, as representative of the kinematic:


(5) $$B\,P=\min_{t\to F}\left(\left|B\,P_{t}-\frac{\sum_{t=1}^{F}B\,P_{t}}{F}\right|\right)$$

**FIGURE 1:**
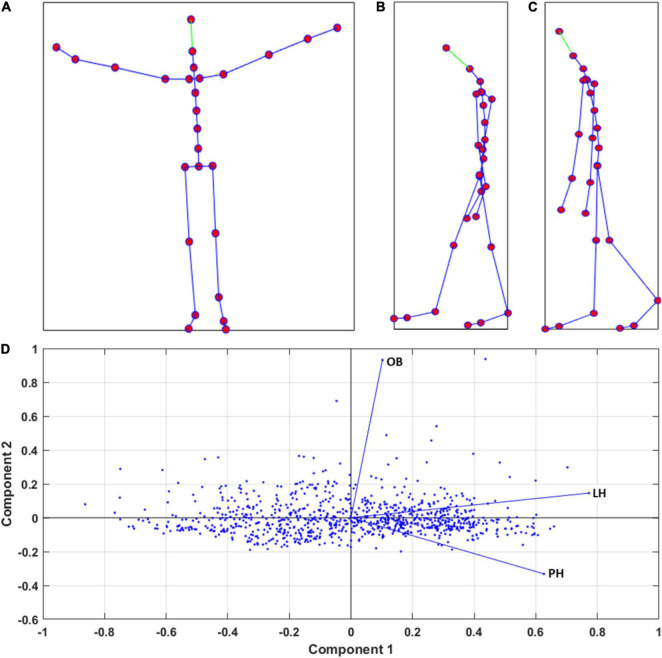
Panels **(A–C)** show an example of body joints configurations (red dots) depicting a characteristic body posture for the body features OB, LH, and PH, respectively. Panel **(D)** displays the distribution of the 912 simple walking actions (blue dots) described by a particular score of OB, LH, and PH, over the plane composed by the Component 1 and Component 2 derived from the PCA.

Figure 1 shows the body-joint configurations representing the body features adopted to compute the BP, i.e., the LH, PH, and OB, and illustrates the distribution of the 912 walking actions in the plane defined by the first two components of the PCA, according to their value of LH, PH, and OB.

### Body Dynamicity

**FIGURE 2:**
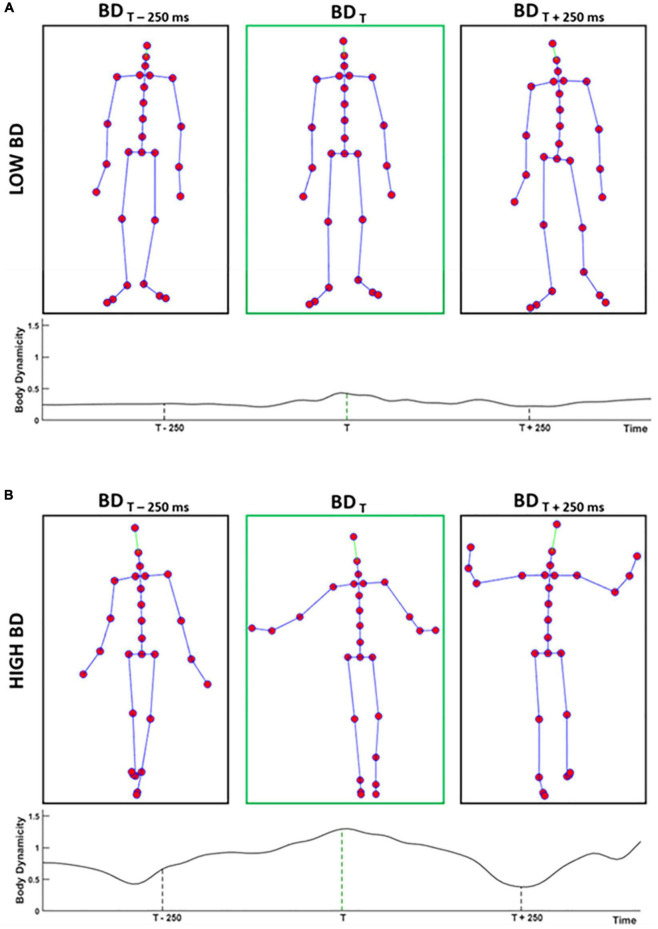
Panels **(A,B)** show three body joints configurations depicting body postures with low (high) level of BD extracted from two different walkings. The BD time course of the walking is reported below each panel, which shows the precise timepoint from which postures are extracted. Middle panels in green illustrate the representative frames of the whole kinematic, extracted according to the procedure described in the Section “Materials and Methods.” Instead, the first and third panels of the two rows represent two frames extracted 250 ms before and 250 ms after the time of the representative BD, respectively.

The dynamicity of the performed action typically contains affective information concerning the arousal dimension (Paterson et al., 2001; Pollick et al., 2001; Nakagawa et al., 2009; Dael et al., 2013). To transfer the dynamicity of the whole kinematic to a static body posture, we defined a BD score and computed this metric for each time frame of the kinematic. We identified the frame associated with the maximum BD value and then selected the corresponding body posture as representative of the arousal level of that action. In detail, for each body joint, we first computed the distance between the position at the time *t* and *t* + 1 divided by the duration of the time frame:


(6) $$B\,D_{j,t}=\,\frac{P_{t+1}-P_{t}}{T_{f}}$$

where *j* and *t* identify the specific body joint and the time frame. To obtain the frame-by-frame BD of the full-body kinematics, we averaged the BD related to the body joints:


(7) $${\bar{B\,D}}_{t}=\,\frac{\sum_{j=1}^{N}B\,D_{j,t}}{N}$$

where *N* is the number of body joints. Finally, we considered the highest BD value across the time frames as representative of the kinematic:


(8) $$B\,D\,=\max_{t\to F}{\bar{B\,D}}_{t}$$

where F stands for the total number of recorded frames for that specific kinematic. Figure 2 shows body joint configurations representing two body postures with low and high levels of BD. Also, the BD time course of the original walking actions is presented.

### Emotional Body Postures

Motion capture data were first converted from.bvh to.fbx file extension using 3ds Max 2020 and following the recommendation described by the Xsens Company.^1^ As explained in the previous sections, for each kinematic, we extracted the two configurations of body joints—representative of body postures—associated with the values of expressed arousal and valence. We then selected 90 body postures in three groups corresponding to the low, middle, and high level of BP, each one counting 15 male and 15 female body postures. Analogously, we selected 90 body postures with low, middle, and high values of BD. We performed two one-way ANOVAs with factors BD [*F*_(2,87)_ = 1197.5, *p* < 0.001] and BP [*F*_(2,87)_ = 1187.4, *p* < 0.001] to assess differences among the levels low, middle, and high of the corresponding emotional dimension. Bonferroni-corrected pairwise comparisons highlighted significant differences among all the three levels of BD and BP. Supplementary Figures 1, 2 illustrate the selection procedure of body postures with different levels of BP and BD, respectively. The corresponding statistical analysis shows that BP and BD levels are balanced between male and female actors. Then, we used these data to animate two humanoid avatars (one male and one female)^2^ exploiting Unity (2019.1.0f2). Here, we covered the avatar’s faces with a skin-colored mask, allowing participants’ responses to depend only on the avatar’s body posture and not on facial expressions.

## Experiment 1

Experiment 1 consisted of two separate online surveys. In Experiment 1.A, participants rated the valence and arousal levels expressed by static body postures. In Experiment 1.B, participants rated the valence and arousal level conveyed by the corresponding walking actions from which the postures were originally extracted.

### Experiment 1.A

#### Stimuli

**FIGURE 3:**
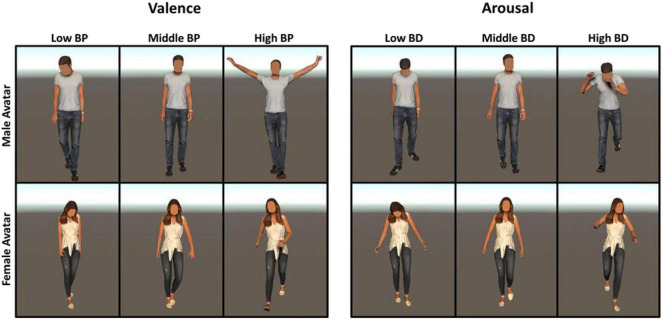
Example of avatar’s body postures with different levels of BP **(Left panels)** and BD **(Right panels)**.

The 180 body postures (90 BP, 90 BD) extracted from actions recorded on male (female) actors were assigned to a male (female) avatar to guarantee coherence between the gender of the actor and that of the virtual avatar. Figure 3 illustrates the representative avatar’s body postures with different levels of BP and BD.

#### Participants, Experimental Procedure, and Data Analysis

We used the online platform Prolific^3^ to recruit participants for the experiment (Palan and Schitter, 2018). To ensure a reliable sample of participants, we recruited only those who reached almost 95% of the approval rate in previous online experiments and declared to speak English fluently. In line with Prolific policy, participants received a payoff (3.20 £) after completing the experiment. A total of 54 age-matched participants [27 women aged 26.3 ± 5.9 years, 27 men aged 23.4 ± 4.3 years; two-sample *t*-test, *t*_(52)_ = −1.366, *p* = 0.177] were recruited for the online survey. The sample size was determined using a power analysis computed through the G*Power software (Faul et al., 2007) considering the “as in SPSS” option and setting the significance level (α) at 0.05, the desired power (1-β) to 0.95, the number of groups to 2, the number of repetition to 6, and the non-sphericity correction ε to 1. As we did not find any previous work related to emotional body perception performed online reporting the values of η_*p*_^2^, we set the η_*p*_^2^ to 0.16, based on this research performed in a laboratory setting (Kret and de Gelder, 2010), and then doubled the estimate sample size, to compensate the limited control on subject attention and accuracy in doing the online experiment.

Our experiment ran on the online platform Pavlovia.^4^ At the beginning of the experiment, participants read written instructions explaining the concepts of valence and arousal and information on how to express their judgments. Valence was described as the pleasantness state expressed by the body posture of the avatar, referring to the positive or negative character of the event that the body is experiencing. Unpleasant states were associated with bad feelings or a negative state of mind, while pleasant states were associated with good feelings or a positive state of mind (Colombetti, 2005). The arousal dimension was described as the state of activation expressed by the body posture of the avatar, representing a change of the individual physical and psychological asset. A deactivated state was associated with a low heartbeat, sweating decrease, slow breathing, absence of energy, and decreased attentional and decisional capability. Instead, an activated state was associated with a high heartbeat, sweating increase, fast breathing, feelings of vigor, energy, tension, and increasing attentional and decisional capability (Kreibig, 2010). In each experimental trial, participants judged the arousal and valence level conveyed by the avatar’s body posture. Specifically, on the left side of the screen, a picture representing an avatar with a specific body posture was presented, while two questions appeared on the right side with which participants could rate the arousal and valence level expressed by the avatar’s body posture. Specifically, as concerns the arousal, they answered the question: “This person looks in a… state” by means of a visual analog scale (VAS) where the lowest value was “deactivated,” numerically associated to 0, and the highest one was “activated,” numerically associated to 1. As concerns valence, they answered the question: “This person looks in a… state” by means of a VAS where the lowest value was “unpleasant,” numerically associated to 0, and the highest one was “pleasant,” numerically associated to 1. Participants gave their judgment by clicking the mouse left button on each rating scale and then pressed the space bar to move to the next trial, thus they had no time limits to answer. The whole experiment comprised 180 trials randomly presented in five separate blocks of 36 trials each. Blocks were separated by a self-paced pause during which participants could rest.

Valence and arousal ratings were normalized between 0 and 1 with the *normalize.m* MATLAB function (method, “range”). This function computes a z-score transformation rescaling changes the distance between the minimum and maximum values in a data set by stretching or squeezing the points along the number line, preserving the shape of the z-score distribution according to the following formula:

$$X_{r\,e\,s\,c\,a\,l\,e\,d}=\,\frac{X-\,m\,i\,n\,X}{m\,a\,x\,X-m\,i\,n\,X}$$

Normalized valence and arousal data complied with a normal distribution as confirmed by Shapiro–Wilk tests (*W* = 0.97 for valence data; *W* = 0.97 for arousal data).

Normalized data were then analyzed *via* two mixed-design ANOVA with avatar gender (male, female) and BP/dynamicity (low, middle, high) as within-subject factors and subject gender (male, female) as between-subject factor.

### Results

#### Valence

**FIGURE 4:**
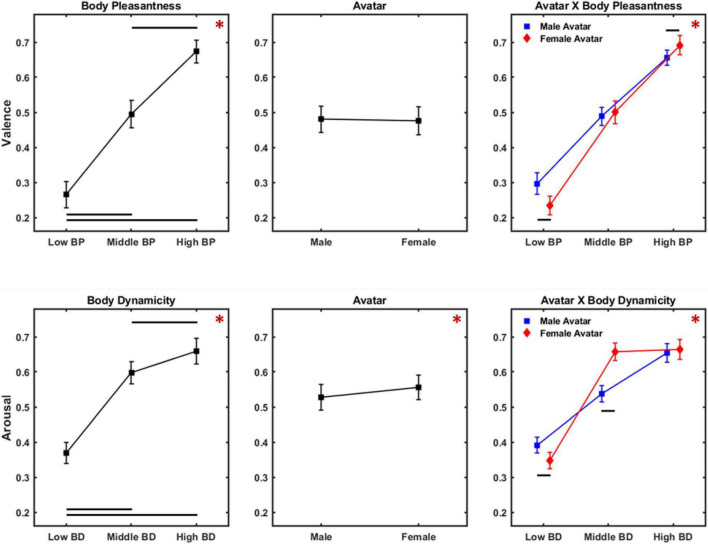
Results of ANOVA on valence **(Upper panels)** and arousal **(Lower panels)** ratings. **(Upper panels)** Present mean and 95% CI of the distribution of the valence scores for the main factors body pleasantness **(Left)**, avatar **(Middle)**, and the interaction avatar × body pleasantness **(Right panel)**. **(Lower panels)** Present mean and 95% CI of the distribution of the arousal scores for the main factors body dynamicity **(Left)**, avatar **(Middle)**, and the interaction avatar × body dynamicity **(Right panel)**. Red asterisks indicate significant main effects, while black lines show significant pairwise comparisons.

Results of the rm ANOVA on valence ratings are illustrated in Figure 4 (upper panels). A significant effect for the main factor BP was found [*F*_(2,104)_ = 476.631, *p* < 0.001, η_*p*_^2^ = 0.902]. Instead, neither the factor avatar [*F*_(1,52)_ = 0.413, *p* = 0.523, η_*p*_^2^ = 0.008] nor subject gender [*F*_(1,52)_ = 0.014, *p* = 0.906, η_*p*_^2^ < 0.001] showed a significant effect on valence ratings. Bonferroni-corrected pairwise comparisons revealed that participants differentially judged avatars whose body postures belonged to different levels of BP (low < middle, *p* < 0.001; middle < high, *p* < 0.001; low < high, *p* < 0.001). Also, the interaction BP × avatar was significant [*F*_(2,104)_ = 35.615, *p* < 0.001, η_*p*_^2^ = 0.406]. Specifically, Bonferroni-corrected pairwise comparison showed that female avatar with low level of BP was judged as less pleasant than male avatar with low level of BP (*p* < 0.001). Also, female avatar with high level of BP was perceived as more pleasant than male ones with high level of BP (*p* < 0.001).

#### Arousal

Figure 4 (lower panels) presents the results of the rm ANOVA on arousal ratings. We found a significant effect for the main factors BD [*F*_(2,104)_ = 383.447, *p* < 0.001, η_*p*_^2^ = 0.881] and avatar [*F*_(1,52)_ = 15.073, *p* < 0.001, η_*p*_^2^ = 0.241]. Conversely, the between-subject factor subject gender did not return a significant effect [*F*_(1,52)_ = 0.367, *p* = 0.547, η_*p*_^2^ = 0.007]. Bonferroni-corrected pairwise comparisons showed that differences among the levels of the main factor BD were all significant (low < middle, *p* < 0.001; middle < high, *p* = 5.3 * 10^–7^; low < high, *p* < 0.001). The significant main factor avatar showed that participants judged female avatar as more arousing than male ones. The interaction body dynamicity × avatar [*F*_(2,104)_ = 101.907, *p* < 0.001, η_*p*_^2^ = 0.662] was also significant, revealing that female avatar with low level of BD was perceived as less arousing compared to male avatar with the same level of BD (*p* < 0.001). Also, female avatar was judged as more arousing than male ones when they belonged to the middle BD level (*p* < 0.001). Finally, we found no significant difference between arousal ratings provided on female avatar with a middle level of BD and female avatar with a high level of BD.

### Experiment 1.B

#### Stimuli

A total of 180 videos were created reproducing the walking actions from which the postures of Experiment 1.A were originally extracted. Walking actions recorded on male (female) actors were used to animate a male (female) avatar, thus ensuring coherence between the gender of the actor and that of the virtual avatar.

#### Participants, Experimental Procedure, and Data Analysis

A total of 22 gender and age-matched participants were recruited and performed the experiment on Pavlovia [11 women aged 29.4 ± 4.3 years, 11 men aged 27.9 ± 4.6 years; two-sample *t*-test, *t*_(52)_ = –0.766, *p* = 0.453].

The adopted experimental procedure was the same as in Experiment 1.A, with the only difference that participants rated the valence and arousal perceived in the emotional walking instead of the corresponding representative static frame. Hence, on the left side of the screen, a video appeared for 2 s showing the walking action, while the two questions were presented on the right side with which participants could rate the arousal and valence level expressed by the avatar’s walking through a VAS.

Valence and arousal ratings were normalized according to the same procedure used in the Experiment 1.A. Then, Pearson’s linear correlation coefficient was computed to assess the correlation between valence (arousal) ratings that participants gave on static body postures and those given on the corresponding walking actions.

### Results

**FIGURE 5:**
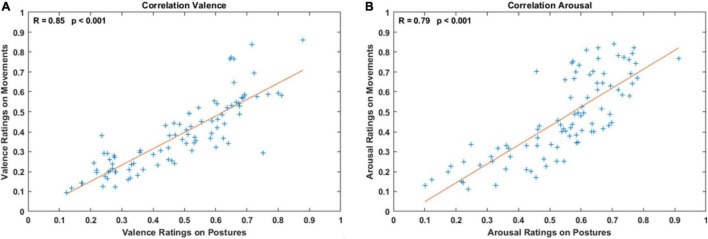
Panels **(A,B)** show the correlation between valence (arousal) scores when subjects rated static postures and the corresponding walking actions. Pearson’s linear correlation coefficient and *p*-values are reported on the top of both panels. Red lines indicate the best linear fit between ratings.

Results of the correlation analysis are illustrated in Figure 5. Panel A shows the positive correlation between valence ratings between the two experiments (*R* = 0.85, *p* < 0.001; best linear fit: *y* = 0.82x − 0.01). Similarly, panel B shows the correlation between arousal ratings (*R* = 0.79, *p* < 0.001; best linear fit: *y* = 0.95x − 0.05).

### Discussion

We defined two body features related to pleasantness and dynamicity based on kinematic and postural information extracted from a set of emotional walking recorded with a motion capture system. We created a male and female avatar with body postures corresponding to three levels of BP and BD, coherently to the gender of the actor on which the kinematics were recorded. Experiment 1.A showed that participants coherently judged the avatar’s bodily valence and arousal according to the defined BP and dynamicity levels. We also investigated whether the gender of the avatar influenced the perception of both valence and arousal, finding that participants perceived female avatar as more emotionally expressive than male ones. Experiment 1.B provided a ground truth comparison showing that valence and arousal levels perceived in the static body postures were consistent with those perceived in the corresponding walking actions.

Overall, we can argue that the defined bodily features allowed the affective information transfer from a full-body kinematic to a static body posture (see Supplementary Material for additional information). The adopted experimental procedure pointed to a difference in perception, possibly due to the avatar’s gender. Despite the existing literature related to the gender difference in affective perception (Codispoti et al., 2008; Samadani et al., 2012), we did not observe any significant difference in the valence and arousal scores between male and female participants.

These results demonstrate that the defined BP, computed as the weighted sum of the openness of the body, leaning of the head, and leaning of the trunk, is a reliable descriptor of the valence dimension (Kleinsmith and Bianchi-Berthouze, 2007; Karg et al., 2013; Fourati and Pelachaud, 2018; Poyo Solanas et al., 2020), being able to transfer the affective information conveyed by an emotional walk to a static body posture. In fact, in this experiment, participants judged the valence level of the avatar coherently to the BP of the posture, distinguishing among low, middle, and high levels. In addition, the female avatar was rated in a more unpleasant state in the low BP condition and more pleasant in the high condition than the corresponding male avatar’s judgments. Thus, participants judged the pleasantness state of the female avatar over a broader range, denoting that these postures were perceived as more expressive when characterized by unpleasant states and when expressing pleasant feelings. This result may reflect the female’s higher emotional expressiveness. Because we normalized the displayed bodily features for the actor’s gender, we may assume that the resulting differences depend on the observer’s perception. Indeed, in line with biological and social models (Eagly and Wood, 1991; Brody and Hall, 2008), women are usually considered more emotionally expressive than men (Kring and Gordon, 1998; Kret and De Gelder, 2012; Chaplin and Aldao, 2013; Deng et al., 2016). When moving toward a virtual world, such biased perception is transferred from humans to virtual characters, thus making the female avatar seem to be more expressive when compared with their male counterpart (DeWester et al., 2009; Zibrek et al., 2015; Bailey and Blackmore, 2017; Yang and Ryu, 2021).

As to the arousal dimension, we defined the BD as a parameter correlating with the velocity of the performed movement. Our results demonstrated that such affective information was transferred to static body postures. Indeed, participants coherently judged the three levels of BD, thus discriminating different arousal levels in the avatar’s body posture. Previous research has shown that features such as velocity, acceleration, and jerk of the movement were highly correlated to the arousal content of emotional gaits, as well as of more specific movement such as drinking and knocking (Paterson et al., 2001; Pollick et al., 2001; Karg et al., 2010; McColl and Nejat, 2014). In automatic affect recognition, the quantity of motion is considered a discriminant factor to distinguish low-arousing movement from high-arousing ones (Castellano et al., 2007). Also, we found that arousal scores were significantly higher for female avatar than for males. Specifically, such biased perception depends on the higher arousal scores that participants gave female avatar with a middle level of BD. Indeed, these scores were comparable to those provided in the high condition. In addition, when compared with the male counterpart, female avatar was perceived as less arousing if characterized by low BD and more arousing if characterized by middle BD. We interpret such results as further evidence of the higher emotional expressivity of women (Grossman and Wood, 1993; Hess et al., 2000; Brody and Hall, 2008). However, this gender characteristic seems to be balanced in the high BD condition where the higher emotional expressivity of women is matched with the higher male tendency to show specific high-arousing emotions such as anger (Chaplin and Aldao, 2013). In fact, we found no differences in scores between male and female avatars in the high BD condition.

These findings reveal that the women’s higher emotional expressiveness may involve both valence and arousal dimension, suggesting that participants perceive women as modulating the intensity of their affective states on a broader range than men.

To understand whether the women’s higher emotional expressiveness could depend on the esthetics characteristics of the avatar used for the experiment or on the methodology we used to create the actual postures they assumed, we conducted a second experiment in which female avatar assumed body postures derived by male actors’ kinematics and *vice versa*, thus creating an incoherent condition. Should participants still perceive female avatar as more emotionally expressive, we could argue that the avatar’s esthetic characteristics (and not postures) mainly modulate the perceived emotional expressiveness. Conversely, should we find that female body postures are perceived more expressive even when assumed by a male avatar, we could conclude that female body postures (and not the avatar’s esthetic) mainly contribute to modulate expressiveness levels.

## Experiment 2

### Stimuli

**FIGURE 6:**
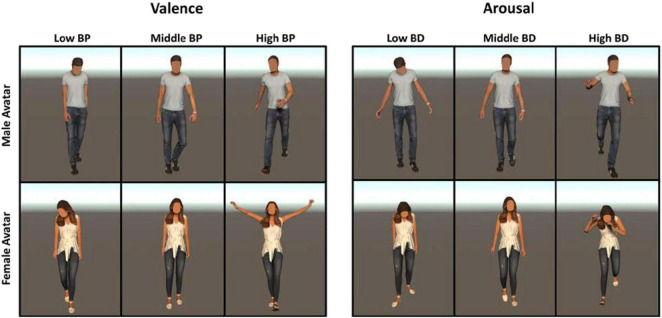
Example of avatar’s body postures with different levels of BP **(Right panels)** and BD **(Left panels)** used in Experiment 2. We assigned the body postures represented in Figure 3 to the gender-opposite avatar.

We used the same body postures of Experiment 1 to animate gender-opposite avatars to produce incoherence between the gender of the avatar and that of the actor from which we extracted the body posture. Hence, body postures extracted from actions recorded on a male actor were assigned to a female avatar and *vice versa*. This procedure led to the creation of 180 stimuli, 90 characterized by different levels of BD and the other 90 by different levels of BP. In Figure 6, we illustrate incoherent stimuli with different levels of BP and BD, showing the same body postures of Figure 3 represented by the gender-opposite avatar.

### Participants, Experimental Procedure, and Data Analysis

The adopted experimental procedure was the same as in Experiment 1. A total of 54 age-matched participants [27 women aged 25.2 ± 5.6 years, 27 men aged 23.4 ± 3.6 years; two-sample *t*-test, *t*_(52)_ = –1.202, *p* = 0.234] were recruited through the online platform Prolific and then performed the experiment on Pavlovia. Also, participants were aged-matched between Experiments 1 and 2 {two-way factorial ANOVA: no significant effect of the main factors subject gender [*F*_(1,104)_ = 3.312, *p* = 0.072] and experiment [*F*_(1,104)_ = 0.793, *p* = 0.375]}.

As in Experiment 1, valence and arousal ratings were normalized between 0 and 1 and then analyzed *via* two mixed-design ANOVA with avatar (male, female) and BP/dynamicity (low, middle, high) as within-subject factors and subject gender (male female) as between-subject factor.

### Results

#### Valence

**FIGURE 7:**
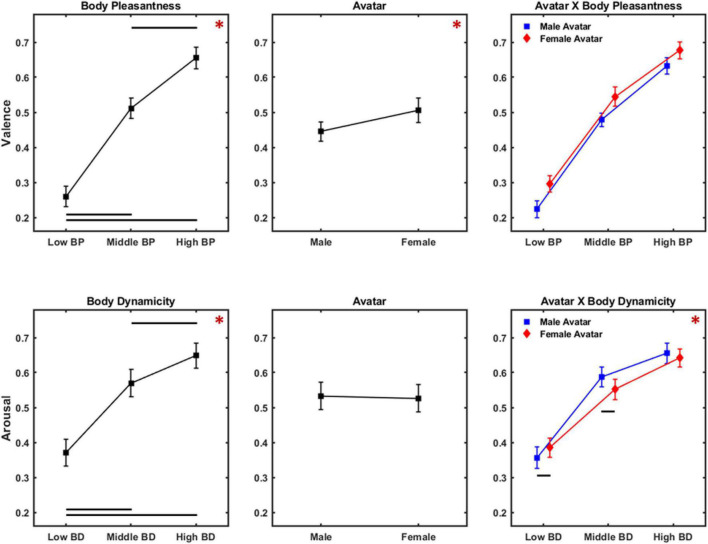
Results of ANOVA on valence **(Upper panels)** and arousal **(Lower panels)** ratings for Experiment 2. **(Upper panels)** Present mean and 95% CI of the distribution of the valence scores for the main factors body pleasantness **(Left)**, avatar **(Middle)**, and the interaction avatar × body pleasantness **(Right panel)**. **(Lower panels)** Present mean and 95% CI of the distribution of the arousal scores for the main factors body dynamicity **(Left)**, avatar **(Middle)**, and the interaction avatar × body dynamicity **(Right panel)**. Same color code as Figure 4.

Figure 7 (upper panels) shows results of rm ANOVA on valence scores. A significant effect for the main factors BP [*F*_(2,104)_ = 570.417, *p* < 0.001, η_*p*_^2^ = 0.916] and avatar [*F*_(1,52)_ = 61.615, *p* = 2.2 * 10^–10^, η_*p*_^2^ = 0.542] emerged. Conversely, no significant effect was observed for the main factor subject gender [*F*_(1,52)_ = 1.857, *p* = 0.178, η_*p*_^2^ = 0.024]. Bonferroni-corrected pairwise comparisons revealed that each level of BP received significantly different scores (low < middle, *p* < 0.001; middle < high, *p* < 0.001; low < high, *p* < 0.001).

#### Arousal

Figure 7 (lower panels) shows the results of rm ANOVA on arousal scores. A significant effect for the main factor BD [*F*_(2,104)_ = 213.137, *p* < 0.001, η_*p*_^2^ = 0.804] was observed, while both factors avatar [*F*_(1,52)_ = 0.891, *p* = 0.349, η_*p*_^2^ = 0.017] and subject gender [*F*_(1,52)_ = 0.031, *p* = 0.860, η_*p*_^2^ < 0.001] did not reveal a significant effect. Bonferroni-corrected pairwise comparisons showed that participants’ ratings were significantly different for each level of the factor BD (low < middle, *p* < 0.001; middle < high, *p* < 0.001; low < high, *p* < 0.001). Also, the interaction avatar × BD [*F*_(2,104)_ = 13.304, *p* < 0.001, η_*p*_^2^ = 0.203] was significant, revealing that female avatar with low BD was perceived as more arousing compared to male avatar with low BD (*p* = 0.026), and that male avatar with middle BD was perceived as more arousing compared to female avatar with middle BD (*p* = 0.002).

### Discussion

In the second experiment, we disrupted the coherence between the actor’s and avatar’s gender to disentangle the relative contribution of body postures and avatars’ esthetic characteristics in judgments on valence and arousal. Findings returned that participants distinguished three levels of valence and arousal expressed by the avatars’ bodies according to the defined BP and dynamicity levels. Such results highlight that even if the avatar assumed postures recorded on actors of the opposite gender, the body features we defined could still transfer the affective information contained in the walking kinematic to a static body posture in terms of valence and arousal. Furthermore, we found that the combination of esthetic characteristics and body postures, i.e., the coherence between the actor’s and avatar’s gender (and not singularly posture or esthetic factor), confers the higher emotional expressiveness to women.

As to the valence ratings, female avatar (with male postures) was perceived in a more pleasant state, regardless of the BP levels to which they belonged. However, considering the interaction between avatar gender and BP, the three levels of the body feature were similarly perceived between female and male avatars. Hence, disrupting the coherence between the gender of the avatar and that of the actor, we found that the avatar/actor gender incoherence spoils the difference between male and female avatars in terms of emotional expressiveness. A recent study reported a similar result using point-light stimuli representing emotional walking. The authors demonstrated that by depriving the participants of the structural cues of the walker gender stimuli were perceived as equally emotionally expressive (Halovic and Kroos, 2018b).

As to arousal, male avatar with body postures extracted from female actresses was perceived in a lower arousing state than female avatar (with male postures) in the low BD condition. On the contrary, participants perceived the female avatar as less arousing than the male one in the middle condition. As in the first experiment, when considering the arousal dimension—regardless of the gender of the avatar—female postures were rated over a broader range compared to male ones. These results testify that the higher female expressiveness revealed in the arousal scores depends on gender-specific kinematic characteristics. These findings align with previous studies, where virtual puppets were more emotionally expressive when animated with female gestures (Yang and Ryu, 2021). However, these outcomes also show the importance of the avatar’s esthetic characteristics, represented by the two genders, for arousal judgments. Participants judged the female body postures differently if applied to a male or female avatar, highlighting that also the gender of the avatar influences the subject’s judgment on arousal perception. In fact, in the second experiment, participants distinguished three levels of BD for female avatar, while in the first one, they confounded the middle with high BD. Similar results also emerged in previous research, showing that the same emotional postures were perceived as more emotionally expressive when represented by female virtual avatars (Zibrek et al., 2015; Cheng et al., 2020).

## Conclusion

This study provides a new method that enables researchers to design body postures of virtual avatars with varying affective states, transferring affective information from dynamic walking to body postures. With this procedure, we created a set of virtual static stimuli potentially useful for studies exploring time-locked cognitive processes such as event-related potentials with electroencephalography, evoked potentials with transcranial magnetic stimulation, and reaction times in all those studies that aim to investigate the perception of emotional body postures.

Two online experiments proved the reliability of the proposed methodology and revealed that male and female avatars are differently perceived when their body posture derives from kinematics recorded on coherent or incoherent gender actors. Therefore, to prevent perceptual biases caused by individual characteristics, it is worth considering the “*actor behind the avatar*” when creating the virtual character with affective postures, i.e., the physical characteristics of the actor when transposing affective information to a virtual avatar. For instance, the height of the actor and his/her size could be relevant information that should also be considered to model the virtual avatar representative of the actor’s affective postures.

Further experiments could extend the validity of the presented methodology considering a more extensive set of kinematics comprising additional emotional gestures other than walking. Finally, exploiting 3D game engine software and virtual reality technologies, this methodology could be used in many experimental settings allowing researchers to resemble different social real-life situations to ultimately reach a deeper comprehension of how we perceive the affective states of others.

## Data Availability Statement

The raw data supporting the conclusions of this article will be made available by the authors, without undue reservation.

## Ethics Statement

The studies involving human participants were reviewed and approved by Comitato Etico AVEN. The patients/participants provided their written informed consent to participate in this study.

## Author Contributions

PP, DR, and GV contributed to the conception and design of the study. PP and GMG collected the data. PP performed the statistical analysis and wrote the first draft of the manuscript. GMG, PA, and FC wrote sections of the manuscript. All authors contributed to manuscript revision, read, and approved the submitted version.

## Conflict of Interest

DR was employed by company TUNED, Lombardini22. The remaining authors declare that the research was conducted in the absence of any commercial or financial relationships that could be construed as a potential conflict of interest.

## Publisher’s Note

All claims expressed in this article are solely those of the authors and do not necessarily represent those of their affiliated organizations, or those of the publisher, the editors and the reviewers. Any product that may be evaluated in this article, or claim that may be made by its manufacturer, is not guaranteed or endorsed by the publisher.

## Footnotes

1
https://www.xsens.com/integrations/3dstudiomax

2 Available on https://renderpeople.com/free-3d-people/.

3
https://www.prolific.co/

4
https://pavlovia.org/
