# Supplementary material for: The Avatar’s Gist: How to Transfer Affective Components From Dynamic Walking to Static Body Postures
Source: Front Neurosci. 2022 Jun 15;16:842433. doi: 10.3389/fnins.2022.842433 (PMC9240741; doi:10.3389/fnins.2022.842433)
Supplement: Supplementary file 1 [file Data_Sheet_1.docx]

**The avatar’s gist: how to transfer affective components from dynamic walking to static body postures**

**Stimuli Selection Procedure**

Nine-hundred-twelve avatar’s body postures were sorted according to their BP (BD) scores. Then, three equidistant groups were identified at the bottom (Low), middle (Middle) and top (High) of the distributions. We avoided selecting postures at the border of the distributions due to the non-linear trend. We discarded by visual inspection very similar postures and finally identified 30 (15 males) postures per condition. To assess differences among the identified groups, we performed two separate 2-way ANOVAs with main factors BP (BD) (Low, Middle, High) and Actor Gender (Male Actor, Female Actress) which represents the gender of the actor from which the posture was extracted. As concerns BP, the main factor Body Pleasantness (F(2, 84) = 1151.3, p < .001) was found to be significant. Bonferroni corrected pairwise comparisons highlighted significant difference across all levels (low < middle, p < .001 ; middle < high, p < .001; low < high, p < .001). The factors Actor Gender (F(1, 84) = .272, p = .603) and the interaction Body Pleasantness x Actor Gender (F(2, 84) = .0419, p = .959) did not return any significant difference. As concern BD, the main factor Body Dynamicity (F(2, 84) = 1170.3, p < .001) was significant. Bonferroni corrected pairwise comparisons highlighted significant difference across all levels (low < middle, p < .001 ; middle < high, p < .001; low < high, p < .001). The factors Actor Gender (F(1, 84) = .572, p = .451), and the interaction Body Dynamicity x Actor Gender (F(2, 84) = .217, p = .805) did not return any significant difference


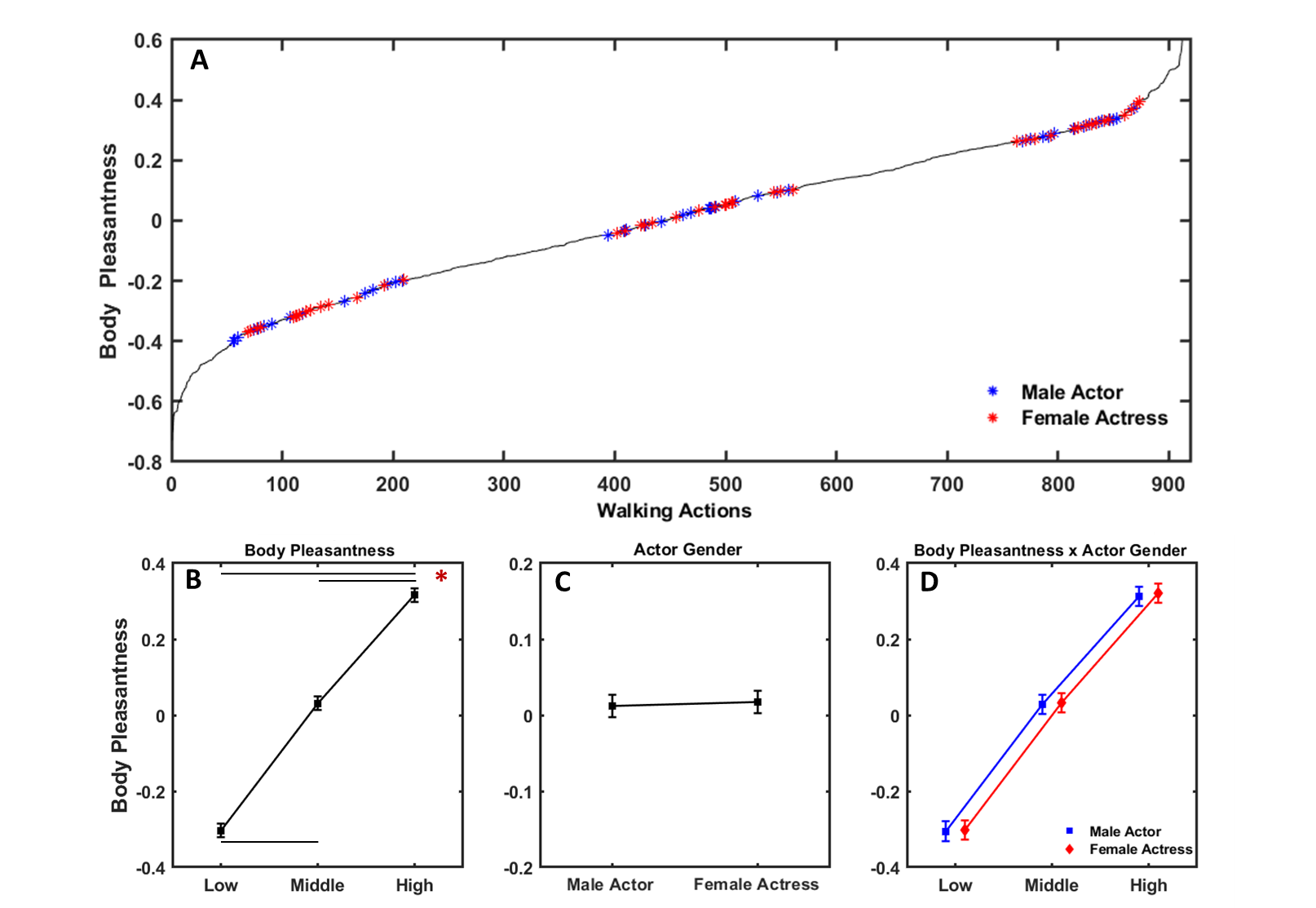


**Fig. S1.** Panel A shows the representative BP scores for all the 912 walking actions. The selected body postures are represented in three separate levels (low, middle, and high) with blue asterisks for male actors and red asterisks for female actresses. Bottom panels represent the results of the 2-way ANOVA computed on the selected BP values. Specifically, panel B presents the significance of the main factor Body Pleasantness (F(2, 84) = 1151.3, p < .001). Bonferroni corrected pairwise comparisons highlighted significant difference across all levels (low < middle, p < .001 ; middle < high, p < .001; low < high, p < .001). The factors Actor Gender (F(1, 84) = .272, p = .603) and Body Pleasantness x Actor Gender (F(2, 84) = .0419, p = .959) did not return any significant difference (panel C and D, respectively).

**
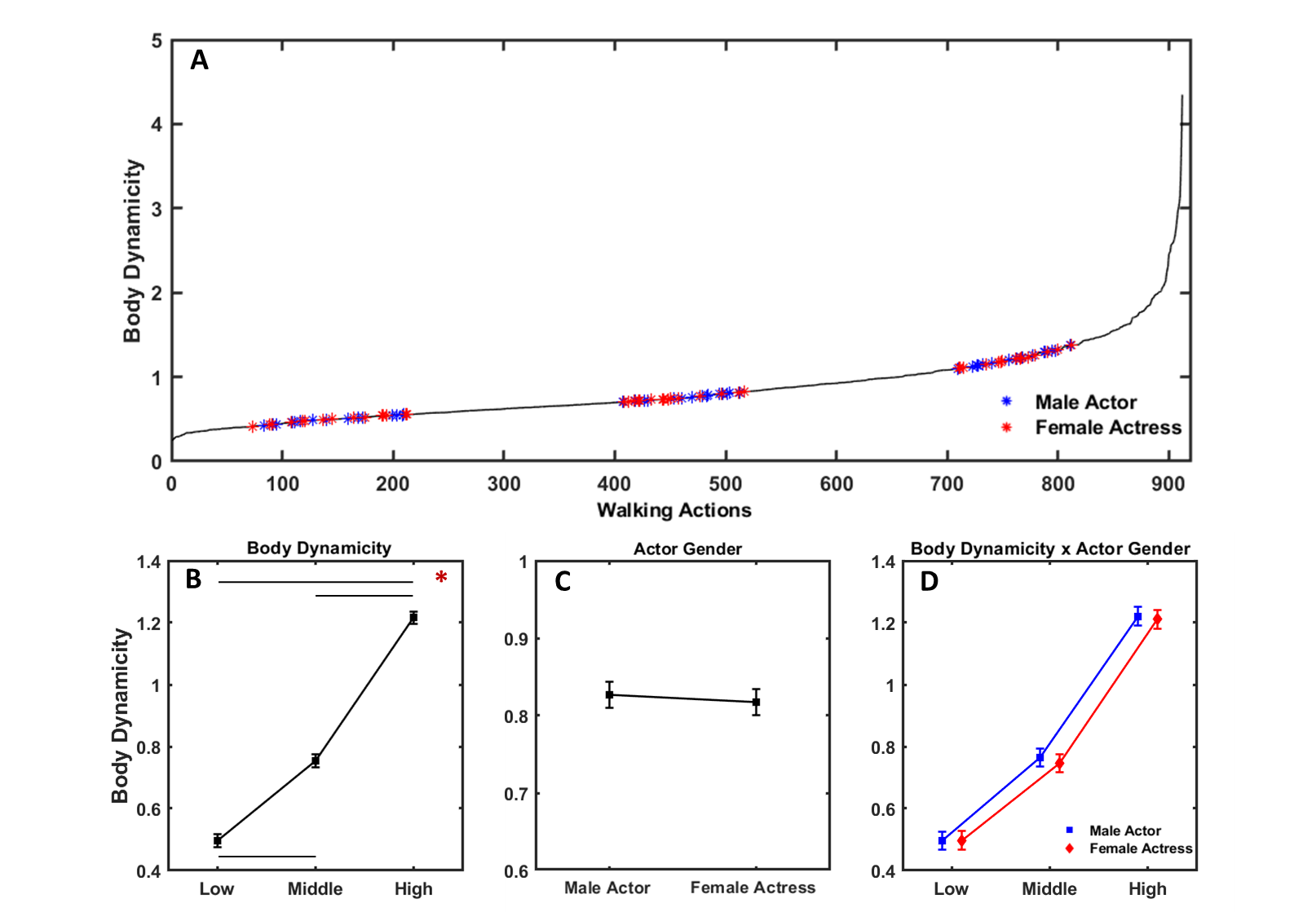
 Fig. S2.** Panel A shows the representative BD scores for all the 912 walking actions. The selected body postures are represented in three separate levels (low, middle, and high) with blue asterisks for male actors and red asterisks for female actresses. Bottom panels represent the results of the 2-way ANOVA computed on the selected BD values. Specifically, panel B presents the significance of the main factor Body Dynamicity (F(2, 84) = 1170.3, p < .001). Bonferroni corrected pairwise comparisons highlighted significant difference across all levels (low < middle, p < .001 ; middle < high, p < .001; low < high, p < .001). The factors Actor Gender (F(1, 84) = .572, p = .451), and Body Dynamicity x Actor Gender (F(2, 84) = .217, p = .805) did not return any significant difference (panel C and D, respectively).

**Definition of valence and arousal as provided in the instructions of the online experiments**

*Arousal*

With the first scale you will judge the state of ACTIVATION expressed by the body posture.

The activation state of the organism is correlated to the change of the individual physical and psychological asset.

A deactivated state is associated with a low heart beat, sweating decrease, slow breathing, absence of energy, decreasing of attentional and decisional capability.

An activated state is associated with a high heart beat, sweating increase, fast breathing, feelings of vigor, energy and tension, increasing of attentional and decisional capability.

*Valence*

With the second scale you will judge the state of PLEASANTNESS expressed by the body posture.

The pleasantness state of a body posture refers to the positive or negative character of the event that the body is experiencing.

An unpleasant state can be associated with bad feelings or negative state of mind.

A pleasant state can be associated with good feelings or positive state of mind.

Press the SPACE BAR to go further.

**Relationships between body cues, and subjective scores**

We performed a correlation analysis considering the bodily indices extracted by the 180 postures used for the experiment to evaluate the relationship between BP and BD. Thus, for each body posture, we considered the BP at the time frame representative for the BD, and vice versa. Pearson’s correlation coefficient was computed for the correlation analysis. As shown in panel A, the figure below illustrates a weak positive correlation between BP and BD scores (R = 0.15, p= 0.04). Then, we evaluated the relation between the subjective ratings of valence and arousal for each body posture, returning a positive correlation (Pearson’s correlation coefficient R = 0.84, p < .001) (see panel B). This result reflects a tendency of participants to judge the avatar’s posture in low arousal and negative valence state or in a high arousal and positive valence state, although the weak correlation between BP and BD. This result is in line with previous theories considering the affect spectrum ranging from low aroused negative affect to highly aroused positive affect (Pettinelli, 2007). This assumption may have its roots in the documented Western preference for highly aroused positive affect (Tsai et al., 2006). From this perspective, the prototype of a positive feeling is excitement (i.e., pleasure accompanied by high arousal), and its opposite is sadness and gloom (i.e., displeasure accompanied by low arousal). Kuppens et al. (2013) report that valence and arousal perceptions can result in six different relationships, regardless of the categorization of the stimuli: independence, a positive linear relation, a negative linear relation, a V-shaped relation, an asymmetrical V-shaped relation, valence as a function of arousal. Although the relation mainly reported in the literature is the V-shaped one, we show that the perception of the stimuli we created falls into one of the main theories describing the relationship between valence and arousal.


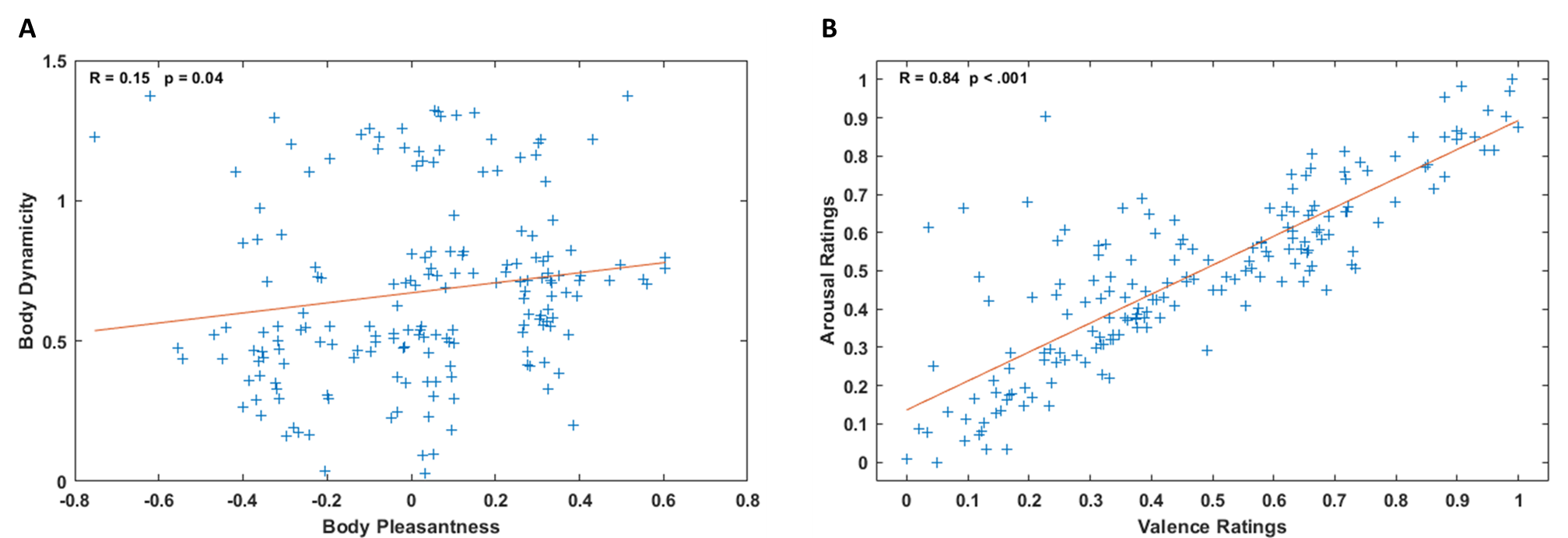


**Fig. S3.** Panel A shows the correlation between BP and BD. Panel B shows the correlation between valence and arousal ratings. valence (arousal) scores when subjects rated static postures and the movements from which they were extracted. The Pearson's linear correlation coefficient and the p-value are reported on the top of both panels. Red lines indicate the best linear fit (Panel A: y = 0.18x + 0.67; Panel B. y = 0.76x + 0.14).

**Reference**

Kuppens, P., Tuerlinckx, F., Russell, J. A., and Barrett, L. F. (2013). The relation between valence and arousal in subjective experience. *Psychol Bull* 139, 917–940. doi:10.1037/a0030811.

Pettinelli, M. (2007). *The Psychology of Emotions, Feelings and.* Gardners Books.

Tsai, J. L., Knutson, B., and Fung, H. H. (2006). Cultural variation in affect valuation. *Journal of Personality and Social Psychology* 90, 288–307. doi:10.1037/0022-3514.90.2.288.
